# Supplementary figures and images for: SANTA: Quantifying the Functional Content of Molecular Networks
Source: PLoS Comput Biol. 2014 Sep 11;10(9):e1003808. doi: 10.1371/journal.pcbi.1003808 (PMC4161294; doi:10.1371/journal.pcbi.1003808)

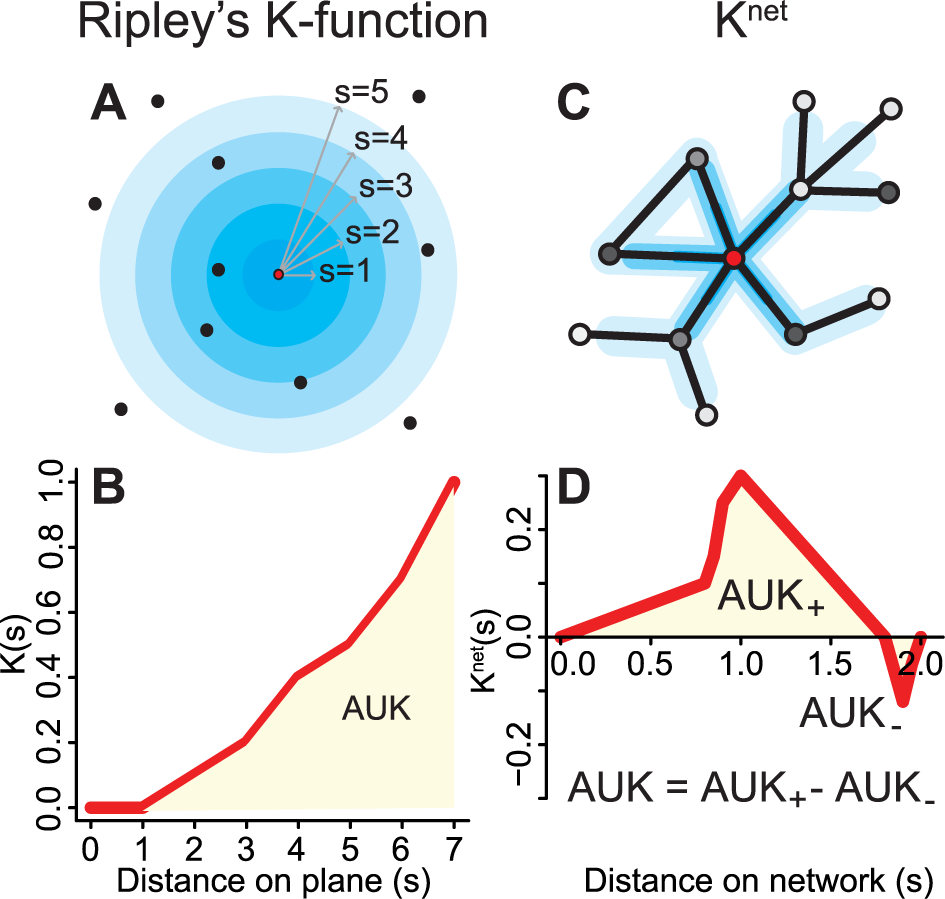

Supplement: Figure S1 — Comparison of Ripley's K-function and . (A) Ripley's K-function () counts how many points on a plane are captured within circles of increasing radius () around each point. Here, circles are drawn from only a single point (red circle). (B) The graph of for the distribution of points in (A). If the clustering of points were greater, then the K(s) function would increase faster and the area under the curve (AUK) would be greater. (C) The -function computes the absolute deviation of the sum of the weight of nodes within a certain distance of each node from the Null model. The distance from a single node (red circle) is shown. The darker the colour of the node, the greater its weight. (D) The graph of the -function for the network and node weights in (C). The greater the clustering of the node weights on the network, the greater the AUK. (TIF) [file pcbi.1003808.s001.tif]

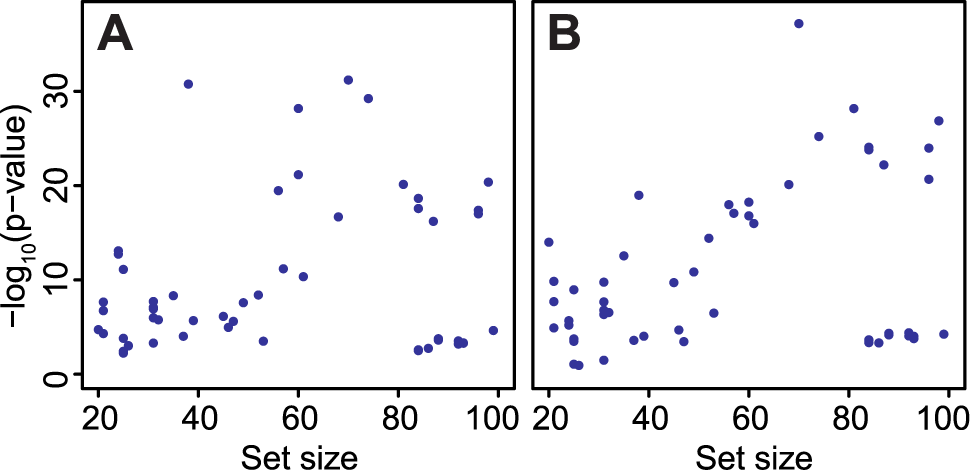

Supplement: Figure S2 — Correlation between set size and p-value. Plot of the significance of the clustering of sets of network genes associated with a GO term against the set size on GI networks mapped in (A) untreated yeast and (B) yeast treated with the DNA-damaging agent MMS. Only those GO terms that associate with either or both networks with a strength of are shown. Many GO terms share a large number of genes due to their ontological relationship. When those GO terms that are ancestors of other GO terms tested are removed, Pearson's correlation coefficient equals 0.004 for the treated network and −0.040 for the untreated network, demonstrating that there is little correlation between set size and p-value. (TIF) [file pcbi.1003808.s002.tif]

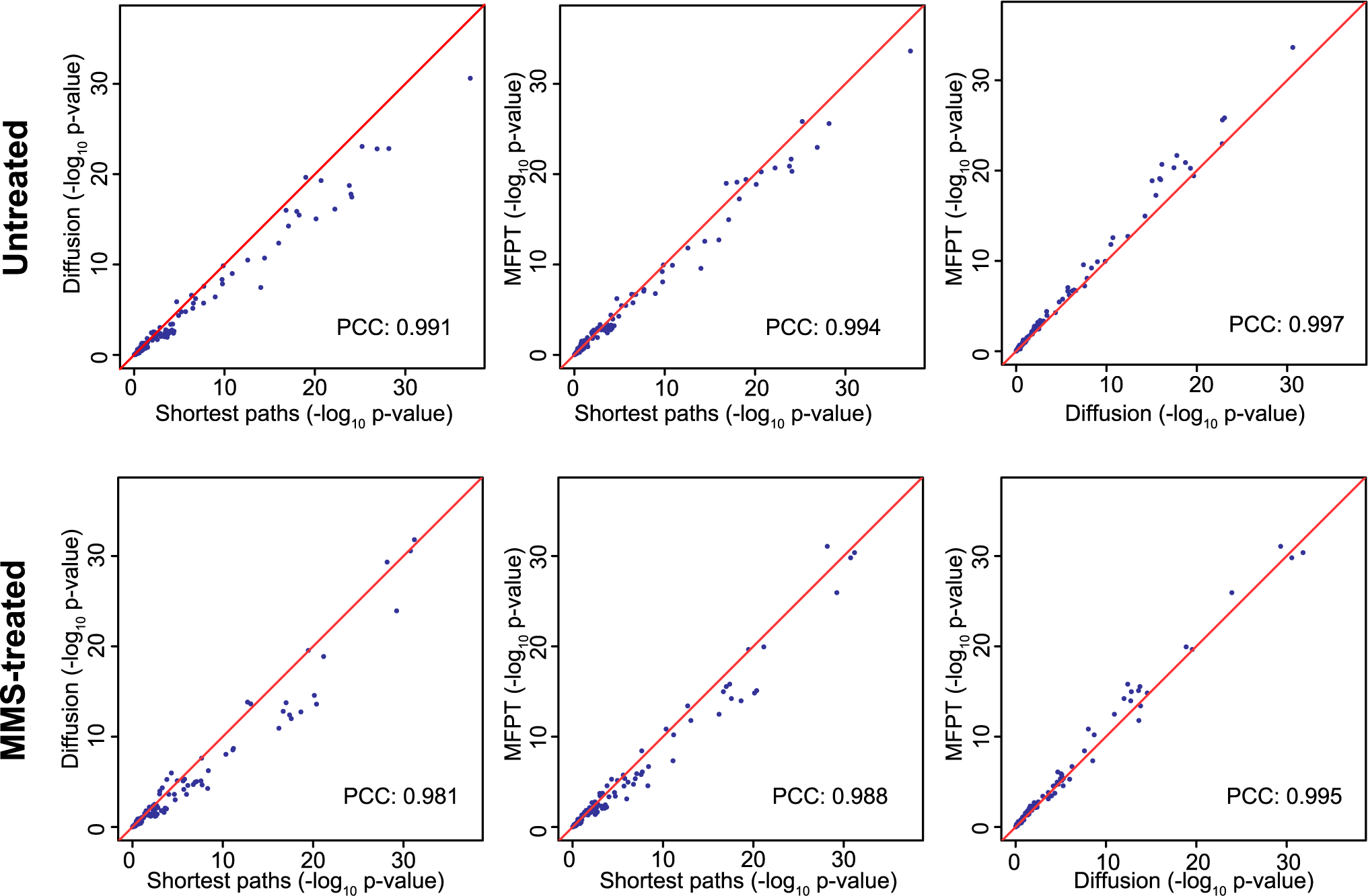

Supplement: Figure S3 — Correlation in network-gene set association strength between distance methods. Pair-wise comparison of the association strengths of GO terms across the three distance methods. The networks tested were the MMS-treated (Top) and untreated (Bottom) S. cerevisiae GI networks created using data from Bandyopadhyay et al. Association strength correlation across networks is very high (), demonstrating that the results produced by SANTA are generally robust across distance methods. (TIF) [file pcbi.1003808.s003.tif]
