# Supplementary material for: SANTA: Quantifying the Functional Content of Molecular Networks
Source: PLoS Comput Biol. 2014 Sep 11;10(9):e1003808. doi: 10.1371/journal.pcbi.1003808 (PMC4161294; doi:10.1371/journal.pcbi.1003808)
Supplement: Table S1 — GO terms differentially associated with a network of raw GIs and GI profile correlations. was used to test the strength of association between sets of genes associated with various GO terms and the two network types. This table contains the GO terms that associated most strongly () with one or both of the networks. GO terms are ranked by their differential association strength (), with the terms associated more strongly with the network of GI profile correlations positioned towards the top and the terms associated more strongly with the network of raw GIs positioned towards the bottom. A greater number of GO term genes associated more strongly with the network of GI profile correlations. (PDF) [file pcbi.1003808.s004.pdf]

Supplementary Table 1. GO terms differentially associated with the network of raw GIs and GI profile correlations

GO Term	p_{raw}	p_{cor}	D	Size
structural constituent of ribosome	3.216e-06	5.366e-46	39.778	100
cytosolic small ribosomal subunit	4.779e-09	2.482e-33	24.285	42
maturation of SSU-rRNA from tricistronic rRNA transcript (SSU-rRNA; 5.8S rRNA; LSU-rRNA)	7.094e-04	4.185e-26	22.229	37
piecemeal microautophagy of nucleus	3.355e-03	1.788e-24	21.273	31
90S preribosome	1.155e-05	4.105e-24	18.449	30
ribosome assembly	1.125e-02	7.359e-21	18.184	23
mRNA splicing; via spliceosome	1.053e-15	7.320e-34	18.158	48
mitotic sister chromatid cohesion	3.523e-12	4.467e-30	17.897	26
mitochondrial respiratory chain	2.741e-05	5.546e-23	17.694	20
ribosomal large subunit biogenesis	2.951e-06	2.614e-23	17.053	31
cytosolic large ribosomal subunit	7.900e-03	7.239e-19	16.038	54
nuclear replication fork	3.886e-11	2.012e-25	14.286	23
mitochondrial membrane organization	2.512e-08	1.370e-22	14.263	30
preribosome; large subunit precursor	1.311e-03	1.636e-17	13.904	22
cytoplasmic microtubule	6.969e-08	7.656e-20	11.959	21
cell division site part	1.060e-07	4.203e-19	11.402	21
rRNA binding	2.483e-02	1.390e-13	11.252	24
regulation of pH	3.710e-05	1.446e-15	10.409	21
tubulin binding	1.100e-13	1.015e-23	10.035	26
DNA-directed RNA polymerase II; holoenzyme	4.162e-08	4.636e-18	9.953	27
mating projection tip	1.541e-12	4.720e-22	9.514	74
U2-type spliceosomal complex	6.987e-08	2.973e-17	9.371	20
CVT pathway	8.063e-04	1.262e-12	8.805	33
oxidative phosphorylation	2.683e-03	5.283e-12	8.706	20
maturation of 5.8S rRNA from tricistronic rRNA transcript (SSU-rRNA; 5.8S rRNA; LSU-rRNA)	3.070e-05	8.746e-14	8.545	27
cellular component movement	1.423e-07	1.887e-15	7.878	21
pre-autophagosomal structure	9.734e-02	2.004e-09	7.686	27
spindle pole body	2.412e-10	2.444e-17	6.994	49
mitochondrial translation	9.621e-09	1.086e-15	6.947	22
small nuclear ribonucleoprotein complex	1.698e-10	2.651e-17	6.807	25
regulation of DNA-dependent DNA replication	1.436e-06	2.339e-13	6.788	22
incipient cellular bud site	4.199e-06	9.896e-13	6.628	41
transcription initiation from RNA polymerase II promoter	1.816e-05	5.915e-12	6.487	22
protein-DNA complex assembly	1.461e-14	1.451e-20	6.003	51
DNA replication initiation	1.331e-10	1.628e-16	5.912	27
mitotic recombination	3.332e-05	7.137e-11	5.669	25
proteasome complex	6.895e-04	1.790e-09	5.586	23
endosome membrane	2.454e-04	6.674e-10	5.566	65
protein complex localization	3.506e-04	1.505e-09	5.367	29
establishment of organelle localization	1.340e-12	1.693e-17	4.898	58
cleavage involved in rRNA processing	3.091e-06	4.239e-11	4.863	24
ER-associated protein catabolic process	1.047e-05	2.565e-10	4.611	36
protein-DNA complex	1.216e-08	3.838e-13	4.501	29
protein heterodimerization activity	9.517e-08	3.372e-12	4.451	23
macroautophagy	2.331e-05	1.906e-09	4.087	40
late endosome to vacuole transport via multivesicular body sorting pathway	4.135e-05	3.701e-09	4.048	21
ER to Golgi vesicle-mediated transport	2.683e-09	3.078e-13	3.940	62
protein import into nucleus	1.322e-10	2.760e-14	3.680	34
chromatin binding	1.425e-08	5.731e-12	3.396	57
condensed nuclear chromosome kinetochore	3.116e-11	2.051e-14	3.182	29
transcription cofactor activity	2.315e-07	2.091e-10	3.044	32
double-strand break repair via homologous recombination	1.100e-11	1.267e-14	2.938	31
nuclear pore	3.408e-10	5.525e-13	2.790	38
intracellular protein transmembrane import	7.411e-07	1.878e-09	2.596	43
regulation of cytoskeleton organization	5.515e-08	1.593e-10	2.539	36
nucleosome organization	6.464e-10	1.995e-12	2.511	45
tRNA wobble uridine modification	2.115e-08	1.493e-10	2.151	20
histone acetyltransferase complex	3.795e-11	3.027e-13	2.098	31
chromatin assembly	7.977e-08	5.181e-09	1.187	30
nuclear chromatin	3.243e-11	4.395e-12	0.868	100
cellular bud tip	2.527e-08	3.858e-09	0.816	77
mRNA export from nucleus	1.050e-09	2.789e-10	0.576	36
spindle organization	2.786e-09	1.666e-09	0.223	22
transcription elongation from RNA polymerase II promoter	1.244e-10	9.306e-10	-0.874	36
cellular response to heat	7.232e-09	4.009e-07	-1.744	29
protein transporter activity	2.648e-09	2.604e-07	-1.993	38
nuclear membrane	8.396e-09	1.825e-05	-3.337	52

Only those GO terms that associate strongly ($p < 1 \times 10^{-8}$) with either or both networks are listed. $D = log_{10}(p_{raw}) - log_{10}(p_{correlation})$. If $D < 0$, then the GO term associates more strongly with the network of raw GIs. If $D > 0$, then the GO term associates more strongly with the network of GI profile correlations. The set size is the number of network genes annotated with the GO term.
