# Supplementary material for: SANTA: Quantifying the Functional Content of Molecular Networks
Source: PLoS Comput Biol. 2014 Sep 11;10(9):e1003808. doi: 10.1371/journal.pcbi.1003808 (PMC4161294; doi:10.1371/journal.pcbi.1003808)
Supplement: Table S2 — GO terms differentially associated with the untreated and MMS-treated GI networks. was used to test the strength of association between sets of genes associated with various GO terms and the two network types. The table contains the GO terms that associated most strongly () with one or both of the networks. GO terms are ranked by their differential association strength (), with the terms associated more strongly with the treated network positioned towards the top and the terms associated more strongly with the untreated network positions towards the bottom. (PDF) [file pcbi.1003808.s005.pdf]

Supplementary Table 2. GO terms differentially associated with the untreated and MMS-treated GI networks

GO Term	$p_{untreated}$	$p_{treated}$	D	Size
DNA repair	1.030e-19	1.714e-31	11.7787	25
response to DNA damage stimulus	1.558e-17	6.604e-29	11.3728	57
covalent chromatin modification	6.059e-06	8.203e-14	7.8684	53
histone modification	2.047e-06	1.852e-13	7.0435	25
chromosome organization	6.043e-26	5.830e-30	4.0155	49
chromatin organization	5.586e-19	6.981e-22	2.9032	45
cell cycle checkpoint	8.511e-02	1.556e-04	2.7378	35
DNA replication	1.080e-09	7.740e-12	2.1447	68
regulation of cell cycle process	3.428e-04	2.481e-06	2.1403	70
regulation of cell cycle phase transition	1.136e-01	9.341e-04	2.0852	25
negative regulation of cell cycle phase transition	1.146e-01	9.950e-04	2.0615	38
chromatin silencing at telomere	1.206e-05	1.834e-07	1.8179	60
negative regulation of cell cycle process	3.214e-02	4.984e-04	1.8094	21
meiotic cell cycle	9.129e-05	2.074e-06	1.6437	31
chromatin modification	1.021e-18	3.421e-20	1.4748	21
regulation of gene expression, epigenetic	4.536e-07	8.062e-08	0.7502	60
meiosis	2.510e-04	9.482e-05	0.4227	25
cell cycle process	5.551e-05	2.312e-05	0.3804	37
macromolecular complex subunit organization	1.993e-05	1.086e-05	0.2636	93
chromatin silencing	1.599e-07	1.172e-07	0.1348	92
negative regulation of gene expression, epigenetic	2.092e-08	1.945e-08	0.0317	47
negative regulation of nitrogen compound metabolic process	6.611e-05	1.718e-04	-0.4148	86
negative regulation of cellular biosynthetic process	1.626e-04	5.199e-04	-0.5049	31
negative regulation of gene expression	4.667e-04	1.818e-03	-0.5907	32
negative regulation of biosynthetic process	9.265e-05	4.724e-04	-0.7074	56
negative regulation of nucleobase-containing compound metabolic process	4.452e-05	2.416e-04	-0.7346	24
gene silencing	2.795e-07	1.698e-06	-0.7835	24
negative regulation of transcription, DNA-dependent	4.342e-04	3.159e-03	-0.8618	99
negative regulation of macromolecule biosynthetic process	8.533e-05	6.318e-04	-0.8695	93
negative regulation of cellular macromolecule biosynthetic process	3.925e-05	2.929e-04	-0.8729	87
negative regulation of RNA metabolic process	2.199e-04	2.581e-03	-1.0694	81
organelle fission	3.156e-04	3.704e-03	-1.0695	31
nuclear division	3.082e-04	4.218e-03	-1.1363	96
mitosis	1.761e-04	5.808e-03	-1.5183	96
chromatin remodeling	1.393e-10	2.229e-08	-2.2041	84
mitotic cell cycle	3.256e-07	3.155e-04	-2.9863	46
chromatin	1.402e-11	2.619e-08	-3.2712	61
protein-DNA complex subunit organization	1.957e-08	4.883e-05	-3.3970	98
chromosome	7.535e-21	2.076e-17	-3.4402	31
nuclear chromatin	1.881e-10	7.399e-07	-3.5947	52
intracellular non-membrane-bounded organelle	2.078e-21	9.921e-18	-3.6788	31
nucleoplasm part	1.691e-10	1.041e-06	-3.7894	84
nucleoplasm	2.737e-13	4.722e-09	-4.2368	88
intracellular organelle lumen	8.280e-25	2.281e-19	-5.4401	25
chromosomal part	1.017e-16	4.537e-11	-5.6496	20
nuclear chromosome	8.379e-18	6.611e-12	-5.8970	88
DNA metabolic process	6.428e-38	6.334e-32	-5.9936	84
membrane-enclosed lumen	6.189e-23	6.189e-17	-6.0000	74
nuclear chromosome part	3.722e-15	3.983e-09	-6.0295	39
organelle lumen	1.566e-24	2.673e-18	-6.2321	84
nuclear part	1.321e-27	4.292e-21	-6.5117	21
non-membrane-bounded organelle	1.024e-24	4.131e-18	-6.6057	26
nuclear lumen	6.551e-29	7.455e-21	-8.0561	26
regulation of DNA metabolic process	9.805e-15	1.875e-05	-9.2815	92

Only those GO terms that associate strongly ($p < 0.001$) with either or both networks are listed.
$D = log_{10}(p_{untreated}) - log_{10}(p_{treated})$. If $D < 0$, then the GO term associates more strongly with the untreated network. If $D > 0$, then the GO term associates more strongly with the MMS-treated network. The set size is the number of network genes annotated with the GO term.
