# Supplementary material for: SANTA: Quantifying the Functional Content of Molecular Networks
Source: PLoS Comput Biol. 2014 Sep 11;10(9):e1003808. doi: 10.1371/journal.pcbi.1003808 (PMC4161294; doi:10.1371/journal.pcbi.1003808)
Supplement: Table S3 — GO terms differentially associated with the untreated and UV-treated GI networks. was used to test the strength of association between sets of genes associated with various GO terms and the two network types. The table contains the GO terms that associated most strongly () with one or both of the networks. GO terms are ranked by their differential association strength (), with the terms associated more strongly with the treated network positioned towards the top and the terms associated more strongly with the untreated network positions towards the bottom. (PDF) [file pcbi.1003808.s006.pdf]

Supplementary Table 3. GO terms differentially associated with the untreated and UV-treated GI networks

GO Term	$p_{untreated}$	$p_{treated}$	D	Size
meiosis I	5.920e-01	2.616e-06	5.355	50
recombinational repair	5.895e-01	3.124e-06	5.276	20
DNA recombination	6.899e-01	4.236e-06	5.212	26
double-strand break repair	1.603e-01	1.090e-06	5.168	87
structure-specific DNA binding	2.033e-01	4.251e-06	4.680	77
transcription factor binding transcription factor activity	1.721e-02	5.523e-06	3.494	61
meiosis	5.581e-01	2.432e-04	3.361	52
nucleotide-excision repair	5.063e-02	2.581e-05	3.293	20
DNA-dependent ATPase activity	9.435e-02	4.940e-05	3.281	31
double-strand break repair via homologous recombination	4.662e-01	2.507e-04	3.269	32
meiotic cell cycle	5.048e-01	5.004e-04	3.004	32
ATPase activity, coupled	1.488e-01	2.004e-04	2.871	48
double-stranded DNA binding	9.225e-03	2.018e-05	2.660	67
transcription cofactor activity	1.744e-01	7.149e-04	2.387	34
helicase activity	9.320e-02	6.406e-04	2.163	41
protein-DNA complex subunit organization	2.031e-03	2.535e-05	1.904	76
SWI/SNF superfamily-type complex	1.056e-03	2.970e-05	1.551	46
negative regulation of transcription from RNA polymerase II promoter	4.932e-03	1.627e-04	1.482	46
protein binding transcription factor activity	1.932e-02	8.910e-04	1.336	24
chromatin remodeling	1.536e-04	5.720e-05	0.429	36
transcription from RNA polymerase I promoter	8.148e-04	4.306e-04	0.277	50
covalent chromatin modification	4.643e-04	6.167e-04	-0.123	90
DNA-dependent transcription, elongation	5.615e-04	1.514e-03	-0.431	37
nucleosome organization	1.041e-05	7.832e-05	-0.877	35
chromatin assembly	8.163e-04	1.174e-02	-1.158	39
histone modification	8.095e-05	2.599e-03	-1.507	77
endosomal transport	9.833e-04	3.323e-02	-1.529	70
DNA packaging	1.223e-05	7.683e-03	-2.798	70
nuclear chromatin	1.772e-09	1.310e-06	-2.869	26
establishment of protein localization to vacuole	1.871e-05	2.347e-02	-3.099	28
chromatin silencing at telomere	1.559e-08	3.392e-05	-3.337	39
vacuolar transport	4.349e-06	2.051e-02	-3.674	76
protein targeting to vacuole	2.582e-06	1.363e-02	-3.722	55
chromatin silencing at silent mating-type cassette	9.137e-07	2.088e-02	-4.359	44
negative regulation of gene expression, epigenetic	6.990e-09	4.060e-04	-4.764	76
chromatin	1.499e-13	1.943e-08	-5.113	93
protein localization to vacuole	5.691e-08	1.360e-02	-5.378	32
chromatin silencing	8.118e-11	2.587e-04	-6.503	71
gene silencing	4.754e-12	2.388e-04	-7.701	37
regulation of gene expression, epigenetic	2.138e-14	4.675e-05	-9.340	36

Only those GO terms that associate strongly ($p < 0.001$) with either or both networks are listed.
$D = log_{10}(p_{untreated}) - log_{10}(p_{treated})$. If $D < 0$, then the GO term associates more strongly with the untreated network. If $D > 0$, then the GO term associates more strongly with the UV-treated network. The set size is the number of network genes annotated with the GO term.
