# Supplementary material for: SANTA: Quantifying the Functional Content of Molecular Networks
Source: PLoS Comput Biol. 2014 Sep 11;10(9):e1003808. doi: 10.1371/journal.pcbi.1003808 (PMC4161294; doi:10.1371/journal.pcbi.1003808)
Supplement: Text S1 — Vignette containing details of how to reproduce the results given in this paper. (PDF) [file pcbi.1003808.s007.pdf]

Vignette for SANTA

Alex Cornish and Florian Markowetz

Introduction

This document contains instructions on how to employ the functions contained within the SANTA package. SANTA builds upon Ripley's K-function, a well-established approach in spatial statistics that measures the clustering of points on a plane, by applying it to networks. Using this approach, the `Knet` and `Knode` functions are defined. `Knet` detects the clustering of hits on a network in order to measure the strength of association between the network and a phenotype. `Knode` ranks genes according their distance from the hits, providing a natural way of prioritising vertices for follow-up analyses.

SANTA addresses two complementary goals: it can (i) functionally annotate experimentally derived networks using curated gene sets, and (ii) annotate experimentally derived gene sets using curated networks.

The SANTA package also contains functions that can be used to measure the distance between vertex pairs (`GraphDiffusion` and `GraphMFPT`) and a function that can be used to visualise the `Knet` function (`plot.Knet`). The 'igraph} package is used to handle the networks.

This vignette, along with the data provided in the SANTA package, are designed to allow the user to reproduce the results shown in the paper Cornish, A. and Markowetz, F. (2014) SANTA: quantifying the functional content of molecular networks. (Under review.) To reduce the time required to run the vignette, many of the case studies are set up to run with a reduced number of trials, permutations or gene sets. It is noted where and how the case studies differ from the paper analyses. The methods used by the SANTA package are further explained in the paper.

Overview of SANTA

The guilt-by-association priciple The guilt-by-association (GBA) principle states that genes that interact are more likely to share biological function. For example, two genes seen to interact in a synthetic genetic array (SGA) are more likely to encode products involved in the same pathway or complex than two genes not seen to interact. `Knet` uses this principle to measure the strength of association between a network and a phenotype. By measuring the clustering of the gene set across biological networks it becomes possible to identify the network that best explains the mechanisms and processes that underlie the set. This principle is also used by `Knode` to prioritise genes for follow up studies. If a set of genes has been identified as being associated with a particular cellular function, then the GBA principle states that genes seen interacting with this set are also likely to be involved in the function.

The `Knet` function The `Knet` function measures the strength of association between a network and a phenotype by quantifying the clustering of high-weight vertices on the network. High-weight vertices represent those genes that are part of a gene set or are most strongly linked with the phenotype. The GBA principle implies that the stronger the clustering, the better the network is at explaining the mechanisms that produce and maintain the phenotype.

The function is based upon Ripley's K-statistic, a tool from spatial statistics that measures the clustering of points on a plane. Ripley's K-statistic works by counting the number of points contained within circles of radius s positioned around each point. The value of s is increased and the area under the resulting curve (AUK) is measured. If points cluster, then they will be engulfed within the circles quicker, the function will increase faster and the AUK will be larger. By swapping the Euclidean distance across the plane with a

measure of distance across the network, this statistic can be adapted for networks. We call this function the `Knet` function.

Unlike Ripley's K-statistic, the `Knet` function can measure the clustering of a set of vertices or a continuous distribution of vertex weights. If biological networks are being used, then a high weight would indicate that a gene is strongly linked with the phenotype being studied. In some scenarios, weights may not be available for all vertices. Vertices with missing weights can be included within the network. However, they should be given a weight of 'NA} to ensure that their distribution does not affect the significance analysis.

The significance of clustering is quantified by permuting the vertex weights across the networks. Vertices with missing weights are excluded. The area under the `Knet` function for the observed set is then compared to the area under the `Knet` function for the permuted sets and a z-test is used to compute a p-value. The p-values produced represents the probability that a clustering of vertex weights at least as strong as that observed occurs given that the vertex weights are distributed randomly across the network.

The `Knet` function is:

$$K^{net}(s) = \frac{2}{(\bar{p}n)^2} \sum_i p_i \sum_j (p_j - \bar{p}) \ \mathbf{I}(d^g(i,j) \leq s)$$

where p_i is the phenotype observed at vertex i, $\mathbf{I}(d^g(i,j) \leq s)$ is an identity function that equals 1 if the distance between vertex i and j is less than s and 0 otherwise, and $\bar{p} = \frac{1}{n}\sum_{i=1}^{n} p_i$.

The `Knode` function Taking the inner sum of the `Knet` equation makes it possible to rank vertices by their distance from high-weight vertices. We call this function the `Knode` function. This function provides a natural way to prioritise genes for follow up studies.

The `Knode` function is:

$$K_i^{node}(s) = \frac{2}{p} \sum_j (p_j - \bar{p}) \ \mathbf{I}(d^g(i,j) \leq s)$$

Measuring the distance between pairs of vertices SANTA contains three methods for measuring the distance between pairs of vertices. Each method incorperates different aspects of the network.

- Shortest paths. The distance along the shortest path connecting two vertices. In a graph without weighted edges, the length of the shortest path is equal to the number of edges in the path. In a graph with weighted edges, the length of the shortest path is equal to the sum of the edge weights in the path. This is the simplist measure of distance and is used by default by the SANTA package.
- Diffusion kernel-based method. The diffusion kernel-based distance measure can be considered as the physical process of diffusion though the graph. Therefore, unlike the shortest paths method, the diffusion kernel-based method incorporates not only the distance along the shortest path connecting two vertices, but the distances along multiple paths. It therefore incorperates more of the network structure.
- Mean first-passage time-based (MFPT) method. The MFPT distance measure computes the distance between vertex i and j ($m_{i,j}$) by computing the expected amount of time that a random walk emanating from node i takes to reach node j for the first time. Much like the difusion kernel-based method, the MFPT method incorperates the number of paths connecting a pair of vertices.

Case studies

i) `Knet` successfully identifies clustering on simulated networks In this section, we will use simulated networks to demnostrate that the `Knet`-function successfully quantifies the clustering of hits on a network.

In order to demonstrate how the `Knet` function can be used to measure the clustering of hits on a network, we will simulate a network, apply clusters of hits of different clustering strengths to the network, and use the `Knet` function to quantify the strength of clustering.

We will use the Barabasi-Albert model of preferential attachment to simulate the networks, as it has been observed that this methods produced networks with attributes similar to many real-world networks. A network of 500 vertices is both large enough to contain clusters of hits of different strengths and small enough to quickly run a large number of trials upon. In the Barabasi-Albert model, vertices are added stepwise and connected to previously-added vertices. This is done under a probability distribution based upon the degree of each vertex. If $power = 1$, then the relationship between the probability distribution and vertex degree is linear. We will use this value, as it creates a network with multiple high-degree hubs, much like many biological networks. Each new vertex is connected to m previously-added vertices. In order to create a moderately-dense network, we will set $m = 1$.

```
# generate the simulated network
require(SANTA)
```

```
## Loading required package: SANTA
## Loading required package: igraph
```

```
require(igraph)
set.seed(1) # for reproducibility
g <- barabasi.game(n=500, power=1, m=1, directed=F)
```

A cluster of hits is simulated by first randomly choosing a seed vertex. All other vertices within the network are then ranked according to their distance to the from the seed vertex. The distance can be measured using any of the previosly described distance metrics (shortest paths, diffusion kernel and MFPT). In this example, we will use the shortest paths metric, as it is the quickest to run. However, running the analysis with the other methods will produce the same results.

```
# measure the distance between pairs of vertices in g
dist.method <- "shortest.paths"
D <- DistGraph(g, dist.method=dist.method, verbose=F)
```

The `Knet` function computes the distances between each pair of verties in g. However, to reduce the time required to run the function, these distances can be computed outside the function. The `Knet` function cannot be run on the raw continuous distances. Therefore, it is neccessary to place the pairs of vertices into discreet bins. This is done using the 'BinGraph} function.

```
# place the distances into discreet bins
B <- BinGraph(D, verbose=F)
```

Next, we will generate clusters of hits and measure the clustering strength using the `Knet` function. The s vertices positioned closest to the seed vertex form the sample set. From this set, 5 vertices are randomly selected to form the hit set. The size of the hit set needs to be small compared to the number of vertices in the network. Increasing the value of s decreases the strength of the clustering. We will test 5 different values of s in order to simulate different clustering strengths. A value of s equal to the total number of vertices in the network produces a hit set containing vertices randomly selected from the entire network. For each value of s, we will conduct 10 trials. In the associated paper, 1000 trials were run. Here we will also use 100 permutations, rather than 1000, in order to reduce the time required.

```
cluster.size <- 5
s.to.use <- c(10, 20, 50, 100, 500)
n.trials <- 10
pvalues <- array(0, dim=c(n.trials, length(s.to.use)),
    dimnames=list(NULL, as.character(s.to.use)))

# run the trials for each value of s
for (s in s.to.use) {
    for (i in 1:n.trials) {
        # generate the hit set
        seed.vertex <- sample(vcount(g), 1) # identify seed
        sample.set <- order(D[seed.vertex, ])[1:s]
        hit.set <- sample(sample.set, cluster.size)

        # measure the stength of association
        g <- set.vertex.attribute(g, name="hits",
            value=as.numeric(1:vcount(g) %in% hit.set))
        pvalues[i, as.character(s)] <- Knet(g, nperm=100,
            dist.method=dist.method, vertex.attr="hits",
            B=B, verbose=F)$pval
    }
}
```

Figure 1 contains the results of the simulation study. The `Knet` function correctly identifies sets of hits created using smaller values of s as clustering more significantly.

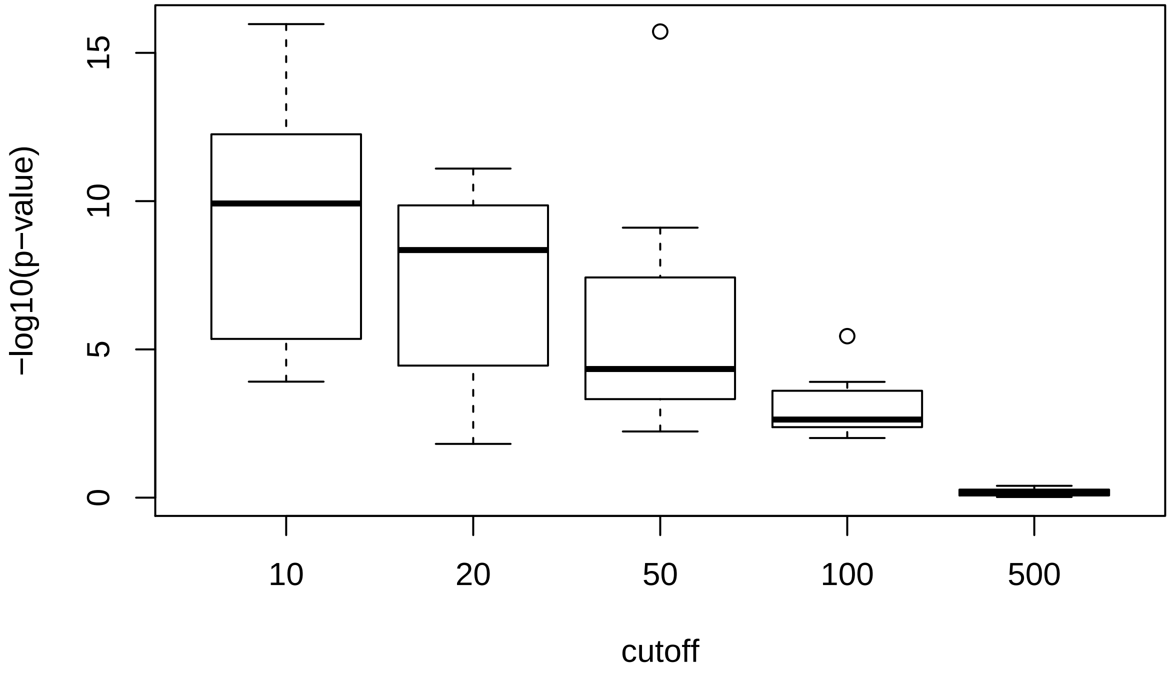

ii) Comparing `Knet` to Compactness Nexy we will demonstrate the effectiveness of the `Knet` function by comparing it to a simpler metric.

Glaab et al. define the compactness score as the mean distance between pairs of hits in a network. By permuting the vertices belonging to the hit set and comparing the observed compactness score to compactness score of the permuted sets, an empirical p-value can be produced. This is much like `Knet`. However, unlike

Knet~, the adapted compactness score cannot be applied to continous distributions of vertex weights and does not incorperate vertex degree when measuring the significance of association. This is demonstrated in this toy example.

First, we create the network. The vertices in this network have a range of degrees, allowing us to demonstrate the differences between Knet and Compactness. We will apply 2 sets of vertex weights, each of which will contain two interacting hits.

```
# create the network
n.nodes <- 12
edges <- c(1,2, 1,3, 1,4, 2,3, 2,4, 3,4, 1,5, 5,6,
           2,7, 7,8, 4,9, 9,10, 3,11, 11,12)
weights1 <- weights2 <- rep(0, n.nodes)
weights1[c(1,2)] <- 1
weights2[c(5,6)] <- 1

g <- graph.empty(n.nodes, directed=F)
g <- add.edges(g, edges)
g <- set.vertex.attribute(g, "weights1", value=weights1)
g <- set.vertex.attribute(g, "weights2", value=weights2)
```

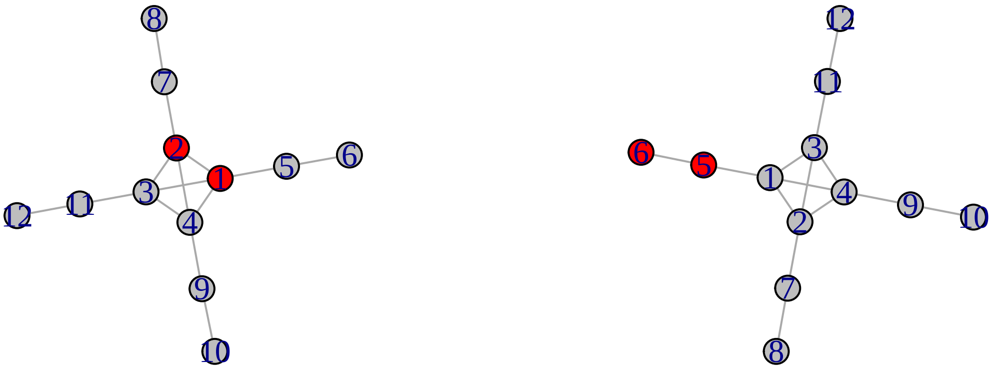

Figure 2 shows the location of each set of hits on the network. The distance between the hits in set 1 and set 2 are the same. However, intuitively we expect that the clustering of the hits in set 2 to be more significant, as the hits have fewer interacting partners and are therefore less likely to be observed interacting by chance.

We will now apply Knet and Compactness to each set of hits in order to measure the clustering significance. We will run the function with 100 permutations. Increasing the number of permutations increases the accuracy of the functions. However, it also increases the run time. 1000 permutations were used to create the results shown in the paper. The shortest paths distance measure will be used. However, other methods will produce the same results.

```
# set 1
Knet(g, nperm=100, vertex.attr="weights1", verbose=F)$pval
```

```
## [1] 0.2984
```

```
Compactness(g, nperm=100, vertex.attr="weights1", verbose=F)$pval
```

```
## [1] 0.1015
```

```r
# set 2
Knet(g, nperm=100, vertex.attr="weights2", verbose=F)$pval
```

```
## [1] 0.003823
```

```r
Compactness(g, nperm=100, vertex.attr="weights2", verbose=F)$pval
```

```
## [1] 0.07638
```

The `Compactness` function does not incorperate vertex degree when measuring the significance of hit clustering. For this reason, it outputs a similar p-value for each hit set. `Knet` incorperates the global distribution of vertices and therefore identifies set 2 as clustering more significanctly than set 1. It is therefore preferable to use `Knet` instead of `Compactness` when the degree of vertices varies across a network.

iii) Using `Knet` to demonstrate that correlation in GI profile produce functionally more informative networks In this section we will demonstrate how the `Knet` function can be used to identify the most effective way of building an interaction network.

There is evidence that protein function is more closely related to global similarity between genetic interaction (GI) profile than to individual interactions. In order to test this, we will use the `Knet` function to quantify the strength of association between GO terms and two S. cerevisiae networks: a network of raw interactions and a network created from corrlelations in interaction profile. Both of these will be created using the data produced by Costanzo et al. If the GO terms associate more strongly with a network, then this is indication that the network is more functionally informative.

First, we will load two pre-processed networks, in the form of `igraph` objects from the `SANTA` package. `SANTA` requires all networks to be in the form of `igraph` objects. The data used to create these networks is available from http://drygin.ccbr.utoronto.ca/~costanzo2009/ and the method used is described in the associated SANTA paper.

`Knet` is also able to incorporate different edge distances when quantifying the clustering of high-weight vertices. Edge distances can represent different biological properties, such as the strength of a physical interaction between 2 gene products, or in the case of our 2 networks, the strength of the genetic interaction. The smaller the edge distance, the stronger or more significant the interaction. When creating the network, we converted the correlation coeficients into edge distances by taking the $-log10$ of the scores. These distances have been stored under the edge attribute `distance`.

```r
# load igraph objects
data(g.costanzo.raw)
data(g.costanzo.cor)
networks <- list(raw=g.costanzo.raw, cor=g.costanzo.cor)
network.names <- names(networks)
network.genes <- V(networks$raw)$name
# genes identical across networks
```

Associations between genes and GO terms will be obtained from the Gene Ontology (GO) project using the `org.Sc.sgd.db` package. In order to reduce the time required to run this analysis, we will use only 5 GO terms. However, it is easy to run this analysis with many more GO terms, as done in the associated paper.

```r
# obtain the GO term associations from org.Sc.sgd.db package
library(org.Sc.sgd.db)
```

```
## Loading required package: AnnotationDbi
## Loading required package: BiocGenerics
## Loading required package: parallel
##
## Attaching package: 'BiocGenerics'
##
## The following objects are masked from 'package:parallel':
##
##     clusterApply, clusterApplyLB, clusterCall, clusterEvalQ,
##     clusterExport, clusterMap, parApply, parCapply, parLapply,
##     parLapplyLB, parRapply, parSapply, parSapplyLB
##
## The following object is masked from 'package:stats':
##
##     xtabs
##
## The following objects are masked from 'package:base':
##
##     anyDuplicated, append, as.data.frame, as.vector, cbind,
##     colnames, do.call, duplicated, eval, evalq, Filter, Find, get,
##     intersect, is.unsorted, lapply, Map, mapply, match, mget,
##     order, paste, pmax, pmax.int, pmin, pmin.int, Position, rank,
##     rbind, Reduce, rep.int, rownames, sapply, setdiff, sort,
##     table, tapply, union, unique, unlist
##
## Loading required package: Biobase
## Welcome to Bioconductor
##
##     Vignettes contain introductory material; view with
##     'browseVignettes()'. To cite Bioconductor, see
##     'citation("Biobase")', and for packages 'citation("pkgname")'.
##
## Loading required package: GenomeInfoDb
## Loading required package: DBI
```

```
xx <- as.list(org.Sc.sgdGO2ALLORFS)
go.terms <- c("GO:0000082", "GO:0003682", "GO:0007265",
              "GO:0040008", "GO:0090329")
# apply the GO terms to the networks
for (name in network.names) {
    for (go.term in go.terms) {
        networks[[name]] <- set.vertex.attribute(
            networks[[name]], name=go.term,
            value=as.numeric(network.genes %in% xx[[go.term]]))
    }
}
```

We will now apply the `Knet` function to each GO terms on each network in order to quantify the strength of association. If a gene is associated with the GO term it will given a weight of 1. If it is not associated, it will be given a weight of 0. We will now store these scores in the 2 graphs under a vertex attribute called `rdds`. Due the time required, the result of this code is commented.

```
# results <- list()
# for (name in network.names) {
#     results[[name]] <- Knet(networks[[name]], nperm=1000,
#         vertex.attr=go.terms, edge.attr="distance", verbose=F)
#     results[[name]] <- sapply(results[[name]],
#         function(res) res$pval)
# }
```

This code can be used to plot the results. Each point represents a single GO term. The $-log_{10}$ of the p-values has been plotted.

```
# p.values <- array(unlist(results), dim=c(length(go.terms),
#       length(network.names)), dimnames=list(go.terms,
#       network.names))
# p.values.ml10 <- -log10(p.values)
# axis.range <- c(0, max(p.values.ml10))
# plot(p.values.ml10[, "raw"], p.values.ml10[, "cor"], asp=1,
#       xlim=axis.range, ylim=axis.range, bty="l",
#       xlab="-log10 of the p-value in the raw GI network",
#       ylab="-log10 of the p-value in the correlation network",
#       main="")
# abline(0, 1, col="red")
```

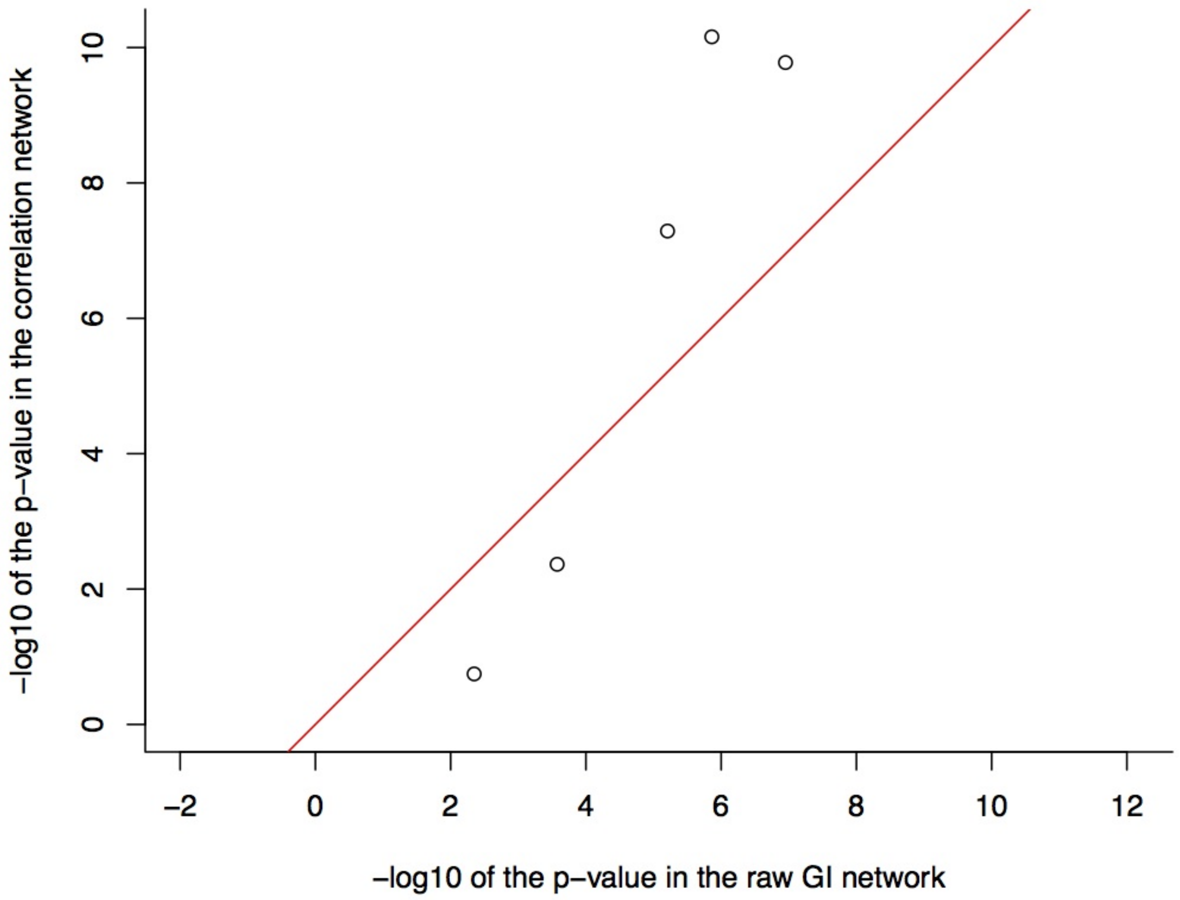

Figure 3 compares the strength of association of each GO term with each network. By increasing the number of GO terms tested, it is possible to demonstrate that using correlation in GI profile rather than raw interaction produces a functionally more informative network.

iv) Using `Knet` to investigate the functional rewiring of the yeast interaction network in response to MMS-treatment In this section we will demonstrate how the `Knet`-function can be used to investigate the functional rewiring of a cell by comparing the strength of association of gene sets between 2 networks.

Bandyopadhyay et al. mapped genetic interaction (GI) networks in yeast under normal laboratory conditions and in yeast exposed to the DNA-damaging agent methyl methanesulfonate (MMS). In order to investigate the differences between these functional networks, we will use the `Knet`-function to quantify the strength of assocation of a set of genes involved in responding to DNA damage. While we cannot learn much from this example, sets of genes involved in other processes can also be tested in order to identify other processes involved in the responce to DNA damage.

We will now load the two interactions networks. These networks were created using correlations in GI profile, as described in the previous section. This is because, as previously demonstrated, using correlations in GI profile as edges results in a functionally more-informative network.

```
# load igraph objects
data(g.bandyopadhyay.treated)
data(g.bandyopadhyay.untreated)
networks <- list(
    treated=g.bandyopadhyay.treated,
    untreated=g.bandyopadhyay.untreated
)
network.names <- names(networks)
```

We will the Gene Ontology (GO) project to identify those genes involved in responding to DNA damage. By changing the code below, it is possible to measure the strength of association of gene sets involved in different functions. In the associated paper, we measured the strength of association of 194 GO terms with each network.

```
# obtain GO term associations
library(org.Sc.sgd.db)
xx <- as.list(org.Sc.sgdGO2ALLORFS)
# change to use alternative GO terms
associated.genes <- xx[["GO:0006974"]]
associations <- sapply(networks, function(g)
    as.numeric(V(g)$name %in% associated.genes),
    simplify=F)
networks <- sapply(network.names, function(name)
    set.vertex.attribute(networks[[name]], "rdds",
    value=associations[[name]]), simplify=F)
```

We will now apply the `Knet` function to the GO term on each network in order to measure the strength of association. The greater the number of permutations run, the greater the accuracy of the p-value.

We will also run `Knet` using the shortest paths method to compute the distance between vertex pairs. However, as shown in the associated paper, the `diffusion` and `mfpt` distance measures produce the same results. This can be demonstrated by replacing the `dist.method` argument below with one of the alternative distance measures. By running this analysis with additional GO terms, the robustness of the `Knet` across different distance measures can be seen.

```
# results <- sapply(networks, function(g) Knet(g, nperm=1000,
#     dist.method="shortest.paths", vertex.attr="rdds",
#     edge.attr="distance"), simplify=F)
# p.values <- sapply(results, function(res) res$pval)
# p.values
```

Since the GO term tested was `response to DNA damage stimulus`, it is not surprising that the gene set associated more significantly with the treated network (as shown by the lower p-value). The yeast exposed to the DNA-damaging agent have activated or upregulated pathways involved in responding to the agent, thereby increasing the strength of the genetic interaction between DNA-damage response-related genes.

We will now visualise the observed and permuted `Knet` curves and AUKs for the GO term on the DNA-damaged and undamaged networks.

```
# plot(results$treated)
# plot(results$untreated)
```

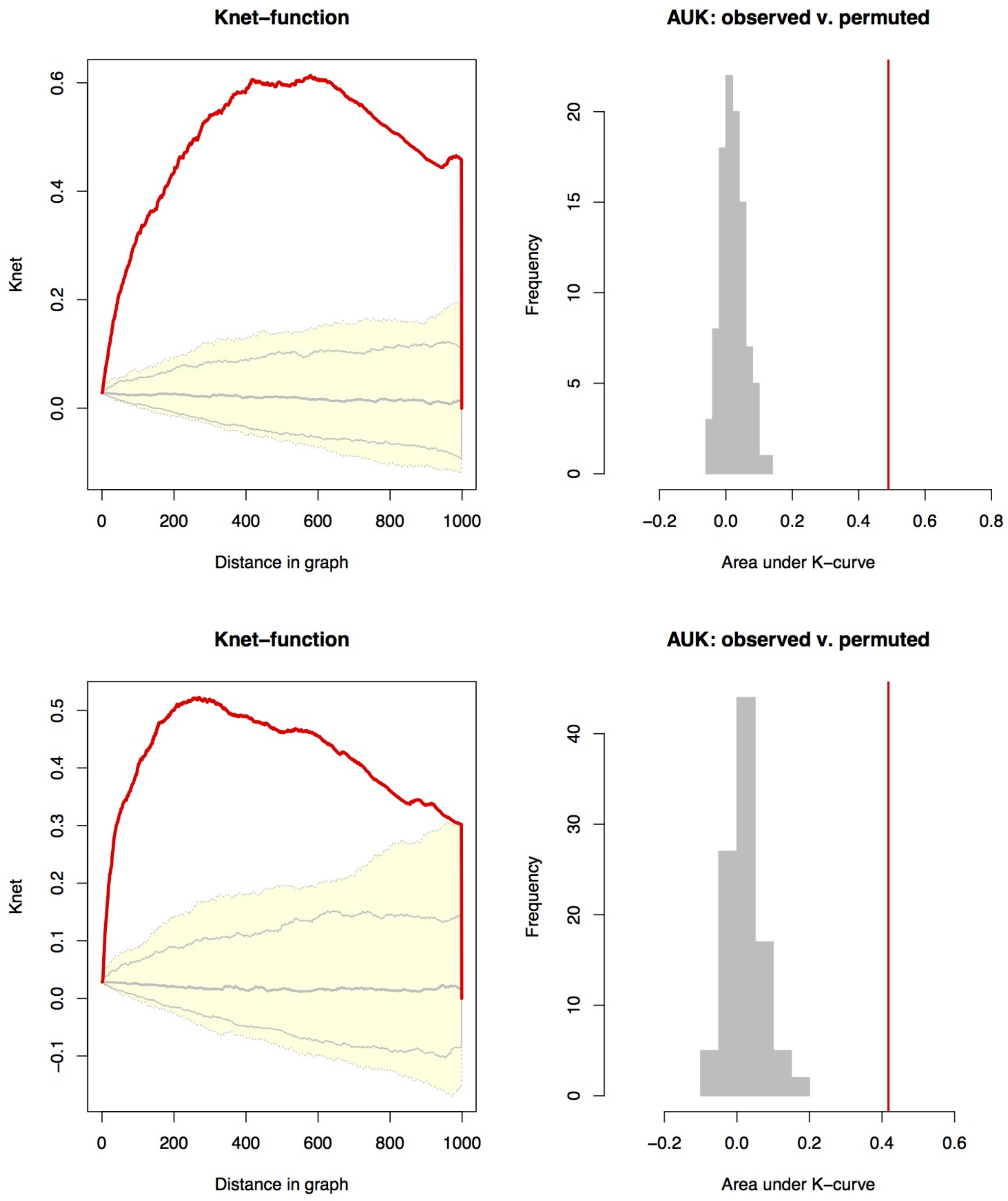

The observed `Knet`-function curve (red line) and the permuted `Knet`-function curve quantiles (yellow area) for the `response to DNA Damage Stimulus` gene set on the DNA-damaged (Top Left) and undamaged (Bottom Left) GI networks. The position of the observe curves relative to the permuted quantiles indicates that the gene set associates strongly with both networks. (Right) The observed `Knet`-function AUK (red line) and the permuted `Knet`-function AUKs (grey histogram) on the DNA-damaged (Top Right) and undamaged (Bottom Right) GI networks. The differences between the observed and permuted AUKs indicates that the genes set

associates strongly with both networks, but most strongly with the DNA-damaged network.

v) Using `Knet` to investigate the functional rewiring of the yeast interaction network in response to UV damage In this section, we will conduct similar analysis to the previous section. However, in this section, we will use a network created from GIs mapped in S. cerevisiae exposed to a high dosage of UV and GIs mapped from untreated S. cerevisiae. Due to the reduced number of interactions mapped in this study, it is not possible to build networks from correlations in GI. Therefore, the networks are built from the raw interactions.

```
# laod igraph object
data(g.srivas.high)
data(g.srivas.untreated)
networks <- list(
    high=g.srivas.high,
    untreated=g.srivas.untreated
)
network.names <- names(networks)
```

We will again test only a single GO term (GO:0000725, recombinational repair). In the associated paper, a greater number of GO terms are tested.

```
# obtain GO term associations
library(org.Sc.sgd.db)
xx <- as.list(org.Sc.sgdGO2ALLORFS)
associated.genes <- xx[["GO:0000725"]]
associations <- sapply(networks, function(g)
    as.numeric(V(g)$name %in% associated.genes),
    simplify=F)
networks <- sapply(network.names, function(name)
    set.vertex.attribute(networks[[name]], "dsbr",
    value=associations[[name]]), simplify=F)
```

The `Knet` function will again be used to compare the strength of association of the GO term with the 2 networks. This code is commented to reduce the time required to run the vignette.

```
# p.values <- sapply(networks, function(g)
#       Knet(g, nperm=1000, dist.method="shortest.paths",
#       vertex.attr="dsbr", edge.attr="distance")$pval)
# p.values
```

As expected, the GO term associates more strongly with the UV treated network. This is likely due to the functional rewiring that occurs within the yeast when exposed to DNA-damaging UV radiation.

vi) Using `Knet` to identify the network most informative about cancer cell lines In this section, we will apply a continuous distribution of vertex weights, describing the strength of evidence that a gene is involved in the maintanence of cancer cell lines, to two networks in order to identify the network that best explains the mechanisms that underlie cell line maintanence.

Biological networks can be built using a wide range of data sources, including co-expression data, data on physical interactions or genetic interaction data. Networks can also be created by combining data sources. The HumanNet network was created by combining 21 data sources. The IntAct network is created using curated data on physical interactions.

In the next section we will apply pre-processed RNAi data from cancer cell lines to both networks as vertex weights. The GENE-E program has been used to convert the essentiality scores for each shRNA into gene-wise p-values. This p-values are contained within the loaded dataset. We will also convert these p-values into vertex weights that can be applied to the networks. By taking the $-log_{10}$ of the p-values, we ensure that the most essential genes are given the highest vertex weights.

```
# import and convert RNAi data
data(rnai.cheung)
rnai.cheung <- -log10(rnai.cheung)
rnai.cheung[!is.finite(rnai.cheung)] <- max(rnai.cheung[is.finite(rnai.cheung)])
rnai.cheung[rnai.cheung < 0] <- 0
```

Next, we will load the interaction data for the two network and create an `igraph` object for each. No edge weights are given for the IntAct network. Each of the interactions in the HumanNet network is associated with a log likelihood score (LLS), describing the probablity the functional interaction occuring given the data used. In order to reduce the density of the network, interactions with an $LLS < 2$ have been removed. Distances for the remaining edges have been produced by dividing the weights by the maximum weight and taking the $-log_{10}$ of this score. We have removed all genes not part of the largest cluster from each network.

```
# import and create IntAct network
data(edgelist.intact)
g.intact <- graph.edgelist(as.matrix(edgelist.intact),
    directed=FALSE)

# import data and create HumanNet network
data(edgelist.humannet)
g.humannet <- graph.edgelist(as.matrix(edgelist.humannet)[,1:2],
    directed=FALSE)
g.humannet <- set.edge.attribute(g.humannet, "distance",
    value=edgelist.humannet$distance)
networks <- list(intact=g.intact, humannet=g.humannet)
```

Next, we will apply the vertex weights to the networks. Not all networks genes have vertex weights available.

```
network.names <- names(networks)
network.genes <- sapply(networks, get.vertex.attribute,
    name="name", simplify=F)
rnai.cheung.genes <- rownames(rnai.cheung)
cancers <- colnames(rnai.cheung)

for (cancer in cancers) {
    for (name in network.names) {
        vertex.weights <-rep(NA, vcount(networks[[name]]))
        names(vertex.weights) <- network.genes[[name]]
        common.genes <- rnai.cheung.genes[rnai.cheung.genes
            %in% network.genes[[name]]]
        vertex.weights[common.genes] <- rnai.cheung[common.genes, cancer]
        networks[[name]] <- set.vertex.attribute(networks[[name]],
            cancer, value=vertex.weights)
    }
}
```

We will now apply the `Knet`-function to these scores. Due to the time required to compute the distance matrices, this code has been commented out. However, it can be easily run if wished.

```
#knet.res <- sapply(networks, Knet, nperm=100,
#   dist.method="shortest.paths", vertex.attr=cancers,
#   edge.attr="distance", simplify=F)
#p.values <- sapply(knet.res, function(i) sapply(i,
#   function(j) j$pval))
```

The RNAi data from the colon and ovarian cancer cell lines associate strongly with both networks. However, they associate associate more strongly with the HumanNet network, indicating that the HumanNet network better explains the cellular mechanisms involved in maintaining these cell lines.

vii) Comparison of Knode and BioNet using simulated data In the next section, we will compare the Knode-function to BioNet. BioNet overlays molecular networks with node information in identifies high-scoring subnetwork. The Knode function identifies nodes close to large numbers of high-scoring nodes and can therefore also be used to identify high-scoring subnetworks. However, as the Knode-function considers each node individually, it can be used to identify high-scoring nodes across multiple clusters.

We will simulate a network containing multiple clusters of high-scoring nodes. We will then apply both the Knode function and BioNet in order to compare performance. In the associated paper, this analysis is repeated using 2, 3 and 4 clusters with 1000 trials. In the code below, only 1 trial is run by default. The code is commented due to the time required.

The BioNet tool is available as an R-package.

```
#library(BioNet)
```

For previously explained reasons, we will use the Barabasi-Albert model of preferential attachment to create the network. We will use the SpreadHits function to apply 3 clusters of 10 hits to the network. In the associated paper, this analysis was also run with 2 and 4 clusters. The SpreadHits function first identifies a seed node for each cluster, located a minumum distance from each other. We will use a distance cutoff of 12, to ensure that each of the clusters of hits is located on a different part of the network. The shortest.paths method will be used to measure distances when spreading hits. However, the same results can be produced using the diffusion and mfpt distance measures. How strongly the hits cluster depend on the parameters lambda, which we will set to 10, to ensure that there is strong clustering of the hits.

```
# # required parameters
# n.nodes <- 1000
# n.hits <- 10
# clusters <- 3
#
# # create network and spread hits across 3 clusters
# g <- barabasi.game(n=n.nodes, power=1, m=1, directed=FALSE)
# g <- SpreadHits(g, h=n.hits, clusters=clusters, distance.cutoff=12,
#     lambda=10, dist.method="shortest.paths", verbose=FALSE)
```

We will now simulate a distribution of p-values and apply them to the network as a vertex attribute. The p-values of the non-hit vertices will be selected from a uniform distribution, as would be expected if there was no associated between these genes and the phenotype being studied. The p-values of the hits will be selected from a truncated normal distribution centered around 0.

```
# # simulate p-values
# library(msm)
# hits <- which(get.vertex.attribute(g, "hits") == 1)
# p.values <- runif(vcount(g))
```

```r
# names(p.values) <- as.character(1:vcount(g))
# p.values[as.character(hits)] <- rtnorm(n.hits * clusters, mean=0,
#     sd=10e-6, lower=0, upper=1)
```

We will now apply BioNet to the network and the simulated p-values, in order to try to identify the enriched subnetworks. A full explanation of the BioNet tool is given in the BioNet R-package vignette. `module` contains each of the vertices identified as being located within an enriched subnetwork.

```r
# # apply BioNet to the network and p-values
# bum <- fitBumModel(p.values, plot=F)
# scores <- scoreNodes(network=g, fb=bum, fdr=0.1)
# module <- runFastHeinz(g, scores)
```

We will now apply the `Knode` function to the network. First, we will transform the p-values to vertex weights. This will be done by taking the $-log_{10}$ of the p-values, in order to ensure that the vertices with the lowest p-values have the greatest scores. `Knode` returns a ranked list of scores for each of the vertices in the network.

```r
# # apply Knode to the network
# g <- set.vertex.attribute(g, name="pheno", value=-log10(p.values))
# knode.results <- Knode(g, dist.method="diffusion",
#     vertex.attr="pheno", verbose=FALSE)
```

In total, 30 hits were applied to the network. We will now compare what number of these 30 hits are contained within the enriched subnetwork identified by BioNet and within the top 30 vertices ranked by the `Knode`-function.

```r
# # number of hits identified by BioNet
# sum(hits %in% as.numeric(V(module)$name))
#
# # number of hits ranked within the top 30 by Knode
# sum(hits %in% as.numeric(names(knode.results)[1:(n.hits * clusters)]))
```

As explained earlier, whilst BioNet tends to identify hits contained only within a single cluster, the fact that `Knode` considers each vertex individually allows it to identify hits contained within multiple clusters. Figure~**??** shows the subnetwork created from only the hits in the network. In the first and second subnetwork, the hits identified by BioNet and Knode are coloured. Vertices successfully identified by BioNet are coloured blue in the left network and those successfully identified by `Knode`~are coloured green on the right network.

```r
# # create subnetworks
# g.bionet <- g.knode <- induced.subgraph(g, hits)
# color.bionet <- color.knode <- rep("grey", vcount(g.bionet))
# color.bionet[hits %in% as.numeric(V(module)$name)] <- "blue"
# color.knode[hits %in% as.numeric(names(knode.results)[1:(n.hits * clusters)])] <- "green"
# g.bionet <- set.vertex.attribute(g.bionet, "color", value=color.bionet)
# g.knode <- set.vertex.attribute(g.knode, "color", value=color.knode)
#
# # plot subnetworks
# par(mfrow=c(1,2))
# plot(g.bionet)
# plot(g.knode)
```

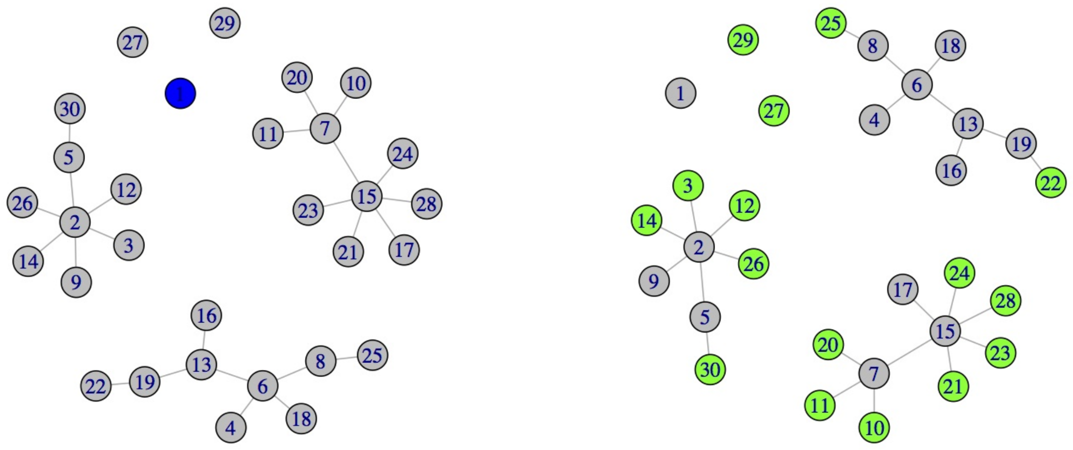

viii) Comparison of `Knode` and BioNet using real data The previous section compared `Knode` and BioNet using simulated data; in this section we will make the comparison using real data. The analysis of this data was originally conducted in the BioNet R-package vignette and discussed in paper by Beisser et al. This makes the comparison of the two methods easier.

In this analysis, we will use expression data from two subtypes of diffuse large B-cell lymphomas (DLBCL, the ABC and GCB subtypes), p-values derived from survival analysis and a network derived from the HPRD database. We will use this data to identify interaction modules involved in DLBCL. This code is commented due to the time required.

```
# # load required package
# library(SANTA)
# library(BioNet)
# library(DLBCL)
# data(exprLym)
# data(dataLym)
# data(interactome)
```

In order to use both the expression and survival data, it is neccessary to combine this into a single p-value. First, differential analysis is used to identify genes that are significantly differentially expression between the two tumour types (results contained within the DLBCL package). We will then use order statistics to combine the differential expression p-values with the survival p-values.

```
# # extract entrez IDs
# library(stringr)
#
# # aggregate p-values
# pvals <- cbind(dataLym$t.pval, dataLym$s.pval)
```

```
# pval <- aggrPvals(pvals, order=2, plot=F)
# names(pval) <- dataLym$label
```

There are a number of genes contained within the HPRD network for which we do not have expression or survival data. We will therefore remove these genes.

The `SANTA` package requires networks to be `igraph` objects. Therefore, we will convert the network from a `graphNEL` object.

```
# # derive Lymphochip-specific network
# network <- subNetwork(featureNames(exprLym), interactome)
# network <- largestComp(network) # use only the largest component
# network <- igraph.from.graphNEL(network, name=T, weight=T)
# network <- simplify(network)
```

We will now use BioNet to identify an enriched subnetwork within the network.

```
# # run BioNet on the Lymphochip-specific network and aggregate p-values
# fb <- fitBumModel(pval, plot=F)
# scores <- scoreNodes(network, fb, fdr=0.001)
# module <- runFastHeinz(network, scores)
# extract.entrez <- function(x) str_extract(str_extract(x,
#   "[(][0-9]+"), "[0-9]+")
# bionet.genes <- extract.entrez(V(module)$name)
```

As previously discussed, the `Knode` function requires p-values to be converted into vertex weights. We will therefore take the $-log_{10}$ of the aggregated p-values and apply these to the network before running the `Knode` function.

```
# # convert p-values to vertex weights
# vertex.weights <- -log10(pval)[get.vertex.attribute(network,
#   "name")]
# network <- set.vertex.attribute(network, name="pheno",
#   value=vertex.weights)
#
# # run Knode on the Lymphochip-specific network and
# # converted aggregate p-values
# knode.results <- Knode(network, dist.method="diffusion",
#     vertex.attr="pheno", verbose=F)
# knode.genes <- extract.entrez(names(knode.results)[1:vcount(module)])
```

Due to the different methods employed by BioNet and `Knode`, different enriched subnetworks are identified. Beisser et al. note that when they apply BioNet to this data, they identify a significant enrichment within the module of genes involved in the regulation of apoptotic process (GO:0042981). Upon application of `Knode`, modules enriched in the regulation of apoptosis are identified ($p < 1 * 10^{-7}$).

```
# data(go.entrez)
# sum(go.entrez %in% bionet.genes)
# sum(go.entrez %in% knode.genes)
```

Session info

This document was produced using:

```r
sessionInfo()
```

```
## R version 3.1.0 (2014-04-10)
## Platform: x86_64-apple-darwin10.8.0 (64-bit)
##
## locale:
## [1] en_GB.UTF-8/en_GB.UTF-8/en_GB.UTF-8/C/en_GB.UTF-8/en_GB.UTF-8
##
## attached base packages:
## [1] parallel  stats     graphics  grDevices utils     datasets  methods
## [8] base
##
## other attached packages:
##  [1] org.Sc.sgd.db_2.14.0 RSQLite_0.11.4       DBI_0.2-7
##  [4] AnnotationDbi_1.26.0 GenomeInfoDb_1.0.2   Biobase_2.24.0
##  [7] BiocGenerics_0.10.0  SANTA_2.0.7          igraph_0.7.1
## [10] formatR_0.10         knitr_1.6
##
## loaded via a namespace (and not attached):
##  [1] digest_0.6.4     evaluate_0.5.5   grid_3.1.0       htmltools_0.2.4
##  [5] IRanges_1.22.9   lattice_0.20-29  Matrix_1.1-4     rmarkdown_0.2.46
##  [9] snow_0.3-13      stats4_3.1.0     stringr_0.6.2    tools_3.1.0
## [13] yaml_2.1.13
```

References

- Sourav Bandyopadhyay, Monika Mehta, Dwight Kuo, Min-kyung Sung, Ryan Chuang, Dorothea Fiedler, Janusz Dutkowski, Aude Gu'{e}nol' e, Haico Attikum, (2010) {Rewiring of genetic networks in response to DNA damage}. *Science* **330** 1385-1390
- Daniela Beisser, Gunnar Klau, Thomas Dandekar, Tobias M"{u}ller, Marcus Dittrich, (2010) {BioNet: an R-Package for the functional analysis of biological networks}. *Bioinformatics* **26** (8) 1129-30 10.1093/bioinformatics/btq089
- Hiu Cheung, Glenn Cowley, Barbara Weir, Jesse Boehm, Scott Rusin, Justine Scott, Alexandra East, Levi Ali, Patrick Lizotte, Terence Wong, Guozhi Jiang, Jessica Hsiao, Craig Mermel, Gad Getz, Jordi Barretina, Shuba Gopal, Pablo Tamayo, Joshua Gould, Aviad Tsherniak, Nicolas Stransky, Biao Luo, Yin Ren, Ronny Drapkin, Sangeeta Bhatia, Jill Mesirov, Levi Garraway, Matthew Meyerson, Eric Lander, David Root, William Hahn, (2011) {Systematic investigation of genetic vulnerabilities across cancer cell lines reveals lineage-specific dependencies in ovarian cancer}. *PNAS* **108** (30) 12372-7 10.1073/pnas.1109363108
- Michael Costanzo, Anastasia Baryshnikova, Jeremy Bellay, Yungil Kim, Eric Spear, Carolyn Sevier, Huiming Ding, Judice Koh, Kiana Toufighi, Sara Mostafavi, Jeany Prinz, Robert P St Onge, Benjamin VanderSluis, Taras Makhnevych, Franco Vizeacoumar, Solmaz Alizadeh, Sondra Bahr, Renee Brost, Yiqun Chen, Murat Cokol, Raamesh Deshpande, Zhijian Li, Zhen-Yuan Lin, Wendy Liang, Michaela Marback, Jadine Paw, Bryan-Joseph San Luis, Ermira Shuteriqi, Amy Tong, Nydia Dyk, Iain Wallace, Joseph Whitney, Matthew Weirauch, Guoqing Zhong, Hongwei Zhu, Walid Houry, Michael Brudno, Sasan Ragibizadeh, Bal'{a}zs Papp, Csaba P'{a}l, Frederick Roth, Guri Giaever, Corey Nislow, Olga Troyanskaya, Howard Bussey, Gary Bader, Anne-Claude Gingras, Quaid Morris, Philip Kim, Chris Kaiser, Chad Myers, Brenda Andrews, Charles Boone, (2010) {The Genetic Landscape of a Cell}. *Science* **327** (5964) 425-31 10.1126/science.1180823

- Carlo Gaetan, Xavier Guyon, (2010) {Spatial Statistics and Modeling}.
- Insuk Lee, U Blom, Peggy Wang, Jung Shim, Edward Marcotte, (2011) {Prioritizing candidate disease genes by network-based boosting of genome-wide association data}. *Genome Research* **21** 1109-21 10.1101/gr.118992.110.7
- Sandra Orchard, Mais Ammari, Bruno Aranda, Lionel Breuza, Leonardo Briganti, Fiona Broackes-Carter, Nancy Campbell, Gayatri Chavali, Carol Chen, Noemi Del-Toro, Margaret Duesbury, Marine Dumousseau, Eugenia Galeota, Ursula Hinz, Marta Iannuccelli, Sruthi Jagannathan, Rafael Jimenez, Jyoti Khadake, Astrid Lagreid, Luana Licata, Ruth Lovering, Birgit Meldal, Anna Melidoni, Mila Milagros, Daniele Peluso, Livia Perfetto, Pablo Porras, Arathi Raghunath, Sylvie Ricard-Blum, Bernd Roechert, Andre Stutz, Michael Tognolli, Kim Roey, Gianni Cesareni, Henning Hermjakob, (2014) {The MIntAct project–IntAct as a common curation platform for 11 molecular interaction databases}. *Nucleic Acids Research* **42** (1) D358-63 10.1093/nar/gkt1115
- Suraj Peri, J Navarro, Ramars Amanchy, Troels Kristiansen, Chandra Jonnalagadda, Vineeth Surendranath, Vidya Niranjan, Babylakshmi Muthusamy, T Gandhi, Mads Gronborg, Nieves Ibarrola, Nandan Deshpande, K Shanker, H Shivashankar, B Rashmi, M Ramya, Zhixing Zhao, K Chandrika, N Padma, H Harsha, a Yatish, M Kavitha, Minal Menezes, Dipanwita Choudhury, Shubha Suresh, Neelanjana Ghosh, R Saravana, Sreenath Chandran, Subhalakshmi Krishna, Mary Joy, Sanjeev Anand, V Madavan, Ansamma Joseph, Guang Wong, William Schiemann, Stefan Constantinescu, Lily Huang, Roya Khosravi-Far, Hanno Steen, Muneesh Tewari, Saghi Ghaffari, Gerard Blobe, Chi Dang, Joe Garcia, Jonathan Pevsner, Ole Jensen, Peter Roepstorff, Krishna Deshpande, Arul Chinnaiyan, Ada Hamosh, Aravinda Chakravarti, Akhilesh Pandey, (2003) {Development of human protein reference database as an initial platform for approaching systems biology in humans}. *Genome Research* **13** (10) 2363-71 10.1101/gr.1680803
- Rohith Srivas, Thomas Costelloe, Anne-Ruxandra Carvunis, Sovan Sarkar, Erik Malta, Su Sun, Marijke Pool, Katherine Licon, Tibor Welsem, Fred Leeuwen, Peter McHugh, Haico Attikum, Trey Ideker, (2013) {A UV-induced genetic network links the RSC complex to nucleotide excision repair and shows dose-dependent rewiring.}. *Cell Reports* **5** (6) 1714-24 10.1016/j.celrep.2013.11.035
- Scott White, Padhraic Smyth, (2003) {Algorithms for estimating relative importance in networks}. *Proceedings of the Ninth ACM SIGKDD International Conference on Knowledge Discovery and Data Mining* 266-75 http://dl.acm.org/citation.cfm?id=956782
